# Machine learning evaluation of pneumonia severity: subgroup performance in the Medical Imaging and Data Resource Center modified radiographic assessment of lung edema mastermind challenge

**DOI:** 10.1117/1.JMI.12.5.054502

**Published:** 2025-10-07

**Authors:** Karen Drukker, Samuel G. Armato, Lubomir Hadjiiski, Judy Gichoya, Nicholas Gruszauskas, Jayashree Kalpathy-Cramer, Hui Li, Kyle J. Myers, Robert M. Tomek, Heather M. Whitney, Zi Zhang, Maryellen L. Giger

**Affiliations:** a The University of Chicago, Department of Radiology, Chicago, Illinois, United States; b University of Michigan, Department of Radiology, Ann Arbor, Michigan, United States; c Emory University, Department of Radiology & Imaging Sciences, Atlanta, Georgia, United States; d University of Colorado Anschutz, Department of Ophthalmology, Aurora, Colorado, United States; e Puente Solutions, LLC, Phoenix, Arizona, United States; f Jefferson Einstein Hospital, Department of Radiology, Philadelphia, Pennsylvania, United States

**Keywords:** artificial intelligence reliability, computer-aided decision support, chest X-ray

## Abstract

**Purpose:**

The Medical Imaging and Data Resource Center Mastermind Grand Challenge of modified radiographic assessment of lung edema (mRALE) tasked participants with developing machine learning techniques for automated COVID-19 severity assessment via mRALE scores on portable chest radiographs (CXRs). We examine potential biases across demographic subgroups for the best-performing models of the nine teams participating in the test phase of the challenge.

**Approach:**

Models were evaluated against a nonpublic test set of CXRs (814 patients) annotated by radiologists for disease severity (mRALE score 0 to 24). Participants used a variety of data and methods for training. Performance was measured using quadratic-weighted kappa (QWK). Bias analyses considered demographics (sex, age, race, ethnicity, and their intersections) using QWK. In addition, for distinguishing no/mild versus moderate/severe disease, equal opportunity difference (EOD) and average absolute odds difference (AAOD) were calculated. Bias was defined as statistically significant QWK subgroup differences, or EOD outside [−0.1; 0.1], or AAOD outside [0; 0.1].

**Results:**

The nine models demonstrated good agreement with the reference standard (QWK 0.74 to 0.88). The winning model (QWK = 0.884 [0.819; 0.949]) was the only model without biases identified in terms of QWK. The runner-up model (QWK = 0.874 [0.813; 0.936]) showed no identified biases in terms of EOD and AAOD, whereas the winning model disadvantaged three subgroups in each of these metrics. The median number of disadvantaged subgroups for all models was 3.

**Conclusions:**

The challenge demonstrated strong model performances but identified subgroup disparities. Bias analysis is essential as models with similar accuracy may exhibit varying fairness.

## 1 Introduction

As COVID-19 transitions to an endemic phase, assessing lung involvement in affected patients remains vital for guiding treatment decisions and managing healthcare resources. Machine learning (ML) algorithms have proven invaluable for tasks such as image segmentation, registration, and computer-aided diagnosis, enabling the identification of patterns in complex medical imaging data that are not easily discernible through human analysis.^1^ These capabilities enhance the accuracy and efficiency of radiologists and other medical professionals.^2^

In the context of COVID-19, AI/ML models have demonstrated their utility in analyzing chest radiographs (CXRs) for automated and consistent assessments of lung damage. The modified radiographic assessment of lung edema (mRALE) score is a widely recognized metric for quantifying lung involvement, facilitating the evaluation of disease severity.^3^^–^^5^ Leveraging AI/ML for mRALE predictions offers a scalable and efficient solution, particularly in high-volume healthcare environments.

The Medical Imaging and Data Resource Center (MIDRC) mRALE Mastermind Challenge aimed to develop AI/ML models to assess COVID-19 severity using mRALE scores (range 0 to 24) derived from portable CXRs. Participants trained their models on various datasets, containerized them using Docker,^6^ and submitted them for evaluation on a hold-out test set inaccessible to participants. Challenge organizers conducted model inference on a computational platform, with detailed performance analyses described in a prior paper.^7^

Although the prior challenge paper focused^7^ on overall model performance, this follow-up study evaluates potential trade-offs between performance and demographic bias in the submitted models. Issues of bias and diversity in AI/ML applications^8^ pose challenges to equitable outcomes in healthcare and in general.^9^^,^^10^ Research has shown that implicit biases in medical imaging can influence diagnostic decisions, potentially leading to disparities based on race, gender, or other demographic factors.^11^ For instance, advanced imaging modalities such as MRI and PET/CT are less accessible in hospitals serving minority populations, contributing to longer wait times and poorer imaging quality.^12^ Biases in AI models often stem from imbalanced training datasets, where over- or under-representation of specific demographic or other groups hinders model generalizability and exacerbates health disparities.^13^ Identifying such biases may be as critical as evaluating overall model performance as biased models may achieve higher performance metrics at the expense of fairness.^14^

Thus, it is essential to rigorously assess AI model performance not just in aggregate but across diverse patient populations, imaging equipment vendors, scanner models, and acquisition protocols. Model generalizability may be compromised not only by inadequate representation in training data but also by technical variability introduced through differences in hardware, software platforms, and clinical workflow. Bias can arise from multiple sources—from systematic differences in imaging equipment or parameters, to site-specific practices—not solely from training data composition.^8^ These factors can perpetuate or even amplify disparities in diagnostic accuracy and risk prediction, especially when AI tools are deployed across varied clinical environments. Therefore, comprehensive evaluation of AI models must account for this spectrum of variables to ensure robust, clinically meaningful performance and minimize unintended consequences across all patient groups and imaging contexts. Although this paper focuses specifically on evaluating performance differences across demographic subgroups, our approach underscores the broader need for ongoing scrutiny of AI model generalizability and potential biases from all sources in medical imaging.

## 2 Material and Methods

### 2.1 Dataset

The MIDRC mRALE Mastermind test set was used to evaluate potential biases in the machine learning models submitted to the MIDRC mRALE Mastermind Challenge. This test set comprised a single portable CXR performed between 1 day prior to and 2 days after a positive PCR test from each of 814 patients, who were categorized into 19 demographic subgroups (Table 1): sex assigned at birth (2 bins), age group (7 bins), race (6 bins), ethnicity (2 bins), and intersectional race and ethnicity (2 bins). Due to the small sample sizes in the “Alaska Native” (N=2 patients) and “Pacific Islander” (N=3) subgroups, these were excluded from bias analysis so that the number of subgroups considered was 17.

**Table 1:** Overview of the test set demographics (total number of patients N=814). Note that not all subgroups for a demographic category may add to 814 because a few “not reported” were not included in the table (N=34, 23, 20, for age, race, and ethnicity, respectively). “Not reported” labels were excluded from bias analysis.

Demographic category	Subgroup	Number of patients
Sex assigned at birth	Female^*^	436 (54%)
	Male	378 (46%)
Age group	Age ≤ 29	145 (18%)
	30 ≤ age ≤ 39	108 (13%)
	40 ≤ age ≤ 49	96 (12%)
	50 ≤ age ≤ 64^*^	192 (24%)
	65 ≤ age ≤ 74	117 (14%)
	75 ≤ age ≤ 84	89 (11%)
	Age ≥ 85	33 (4%)
Race	American Indian or Alaska Native	2 (<1%)
	Asian	31 (4%)
	Black or African American	137 (17%)
	Native Hawaiian or other Pacific Islander	3 (<1%)
	Other	77 (9%)
	White^*^	541 (66%)
Ethnicity	Hispanic or Latino	157 (19%)
	Not Hispanic or Latino^*^	637 (78%)
Intersectional race and ethnicity	White and not Hispanic or Latino^*^	462 (57%)
	Not White or Hispanic or Latino	352 (43%)

* Indicates the largest sub-group for each demographic category used as the reference subgroup in the calculation of bias metrics.

The training dataset, including annotations, is available as described in the “Code and Data Availability” section. The validation and test sets are not yet publicly available.

### 2.2 Evaluated Models

Sixty-three teams registered for this challenge, with ultimately nine participating in the test phase. The best-performing submission of each team (out of a maximum of 3 per team) determined their ranking in the challenge, and those “best” models are the ones evaluated here. Of the nine teams participating in the test phase, five were from the United States, two from Canada, one from Germany, and one from Brazil. Eight were from academic institutions, whereas one was from a governmental agency.

The model code, weights, and descriptions of the models submitted by teams participating in the test phase are available as described in the “Code and Data Availability” section.

### 2.3 Performance Analysis

#### 2.3.1 Estimation task

In the challenge itself, the full estimation problem was evaluated, and the mRALE scores estimated by the participants’ models were compared with the reference standard by means of quadratic-weighted kappa (QWK)^15^ and prediction probability concordance.^16^

#### 2.3.2 Binarized classification task

For the sake of this study, we also investigated the binarized classification problem of distinguishing between no/mild and moderate/severe COVID-19 disease using a threshold value of 4 for the mRALE scores.^5^ To gain insight into model performances in this binarized task, the model-estimated mRALE scores were used as surrogate probabilities of COVID-19 severity, and the area under the receiver operating curve (ROC)^17^ served as the figure of merit.

### 2.4 Bias Measurements

Bias was assessed by analyzing the performance of submitted models across various patient demographics in the challenge test set, both for the estimation task (estimating the reference mRALE score) and for the binarized classification task (distinguishing between no/mild and moderate/severe disease). Biases were evaluated for the five demographic categories: sex assigned at birth, age, race, ethnicity, and intersectional race and ethnicity. Within each category, the largest subgroup was designated as the reference group (indicated with an asterisk in Table 1). Model performance on the remaining 12 demographic subgroups, which had sufficient representation, was compared against their respective reference groups to identify potential biases.

#### 2.4.1 Estimation task

##### Difference in quadratic-weighted kappa

The primary metric for assessing model performance in the challenge was the QWK,^15^ which measures agreement between model output scores (integers on the range [0, 24]) and the reference standard mRALE scores (integers on the range [0, 24]). A statistically significant difference in QWK, ΔQWK, between demographic subgroups, i.e., a 95% confidence interval for ΔQWK that excluded zero (p<0.05) determined via 1000 bootstrap samples, was considered indicative of bias. Given the exploratory nature of this study, no corrections for multiple comparisons were applied.

#### 2.4.2 Binarized classification task

In addition, we calculated the bias metrics of equal opportunity difference (EOD)^18^ and average absolute odds difference (AAOD)^18^ for the binarized task of distinguishing between no/mild and moderate/severe disease. In this task, the reference standard was binarized using a threshold for mRALE to separate no/mild disease (mRALE ≤ 4) and moderate/severe disease (mRALE > 4).^5^ For the calculation of the bias metrics explained below, the same threshold value was applied to the model-estimated mRALE scores as was applied to the reference standard.

##### Equal opportunity difference

EOD measures the difference in true-positive rates (TPRs) between demographic subgroups. Specifically, it quantifies the disparity in the probability that a model correctly identifies a positive case (in this instance, moderate/severe COVID-19 lung disease) for individuals in different subgroups (e.g., race, gender, ethnicity, or age). This metric quantifies whether a model is equally capable of correctly identifying the target condition across subgroups, reducing the risk of unequal access to accurate diagnoses:

$$\mathrm{EOD}=|{\mathrm{TPR}}_{i}-{\mathrm{TPR}}_{j}|,$$ (1)

where “i” and “j” indicate different subgroups. An EOD outside of [−0.1; 0.1] was considered indicative of bias.^19^ *A posteriori* bootstrapping was used (1000 iterations) to estimate the 95% confidence intervals. Subgroups were identified as experiencing bias based on the median EOD obtained from the bootstrap samples.

EOD focuses on TPR, which is important in medical contexts in which misclassification of a positive case (false negatives) could lead to harmful clinical outcomes, e.g., in breast cancer diagnostic workup. Minimizing EOD ensures fairness in the distribution of benefits (e.g., correct treatment).

A potential drawback of this metric is that EOD may be influenced by differences in disease prevalence among subgroups. Although EOD is independent of disease prevalence in a strict mathematical sense—because it only compares the number of true positives to the total number of positive cases—in practice, if a subgroup has a very different prevalence of the disease (i.e., the proportion of actual positive cases differs greatly between groups), it might be easier or harder for a model to maintain the same TPR across groups. A model could appear biased if it performs better in groups where the disease is more prevalent, leading to disparities in TPR between subgroups. Another drawback is that EOD does not consider false positives, so a model could have similar TPRs across groups but differ in false-positive rates, which could result in other forms of bias.

##### Average absolute odds difference

AAOD is a more comprehensive metric that averages disparities in both TPR and false-positive rate (FPR) between demographic groups. It assesses the difference in model performance, accounting for both the ability to correctly identify positive cases (sensitivity) and the rate of incorrect positive identifications (specificity):

$$\mathrm{AAOD}=\frac{1}{2}(|{\mathrm{TPR}}_{i}-{\mathrm{TPR}}_{j}|+|{\mathrm{FPR}}_{i}-{\mathrm{FPR}}_{j}|),$$ (2)

where “i” and “j” indicate different subgroups. An AAOD outside of [0; 0.1] was considered indicative of bias.^19^ *A posteriori* bootstrapping was used (1000 iterations) to estimate the 95% confidence intervals. Subgroups were identified as experiencing bias based on the median AAOD obtained from the bootstrap samples.

AAOD is useful when fairness requires not only equal detection or classification of conditions (as in EOD) but also reducing the burden of false positives, which might lead to unnecessary treatment or additional tests. An advantage of AAOD is that it provides a more balanced view of model fairness by combining both TPR and FPR, reducing bias in multiple aspects of clinical decision-making (both over-treatment and under-treatment). It is less sensitive to prevalence than EOD because it incorporates false positives. However, it can still be influenced by prevalence, e.g., if a condition is less prevalent in a subgroup, there is a higher chance of false positives, which affects FPR and, consequently, AAOD. A drawback is that AAOD involves multiple components, which can make it harder to interpret compared with metrics focused solely on true positives (e.g., EOD). In addition, note that in some medical applications, balancing false positives and true positives may not always be the priority. This can be especially true in high-risk cases where false negatives are of greater concern.

A generalized version of the bias detection code is publicly available, as described in the “Code and Data Availability” section. This code can be applied to any task with an ordinal reference standard and AI output, both of which can be binarized using user-defined thresholds. It allows users to flexibly specify the type and number of relevant population subgroups, including demographics, equipment vendor, image acquisition parameter categories, and more.

## 3 Results

### 3.1 Patient Cohort

**Fig. 1:**
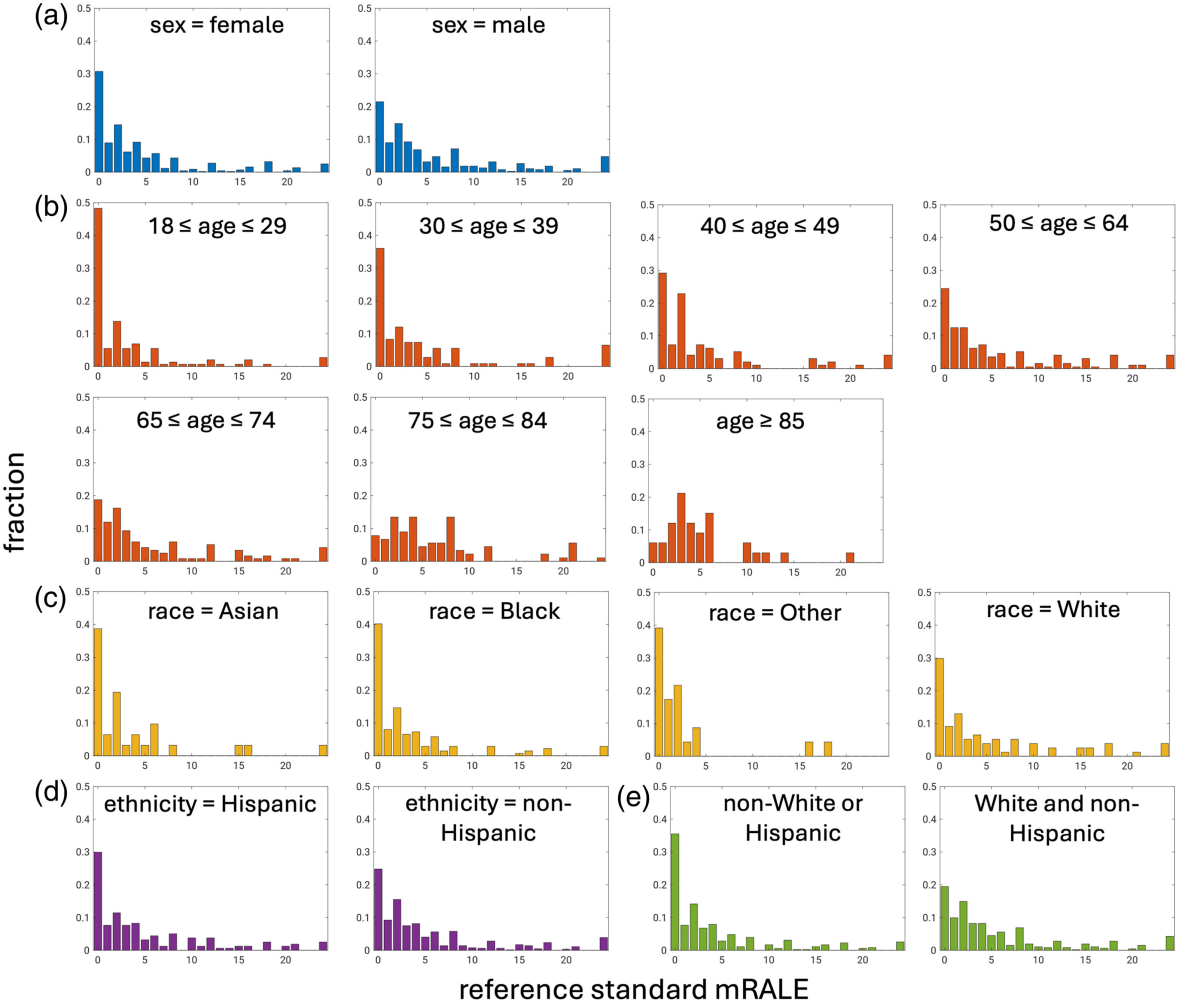
Distributions of reference standard mRALE scores for the different demographic subgroups of (a) sex, (b) age group, (c) race, (d) ethnicity, and (e) intersectional race and ethnicity (Table 1). Note that all distributions have been normalized.

The reference standard mRALE scores for the test set displayed some differences across different demographic subgroups, with older and White patients trending toward more severe disease (Fig. 1). Overall, the prevalences of COVID-19-positive patients without lung involvement (mRALE = 0) and those with no/mild lung involvement (mRALE ≤ 4) were 26% (215/814) and 66% (535/814), respectively.

### 3.2 Model Performance

#### 3.2.1 Estimation task

In the estimation task, the QWK achieved by the models ranged from 0.88 to 0.74, indicating good agreement with the reference standard.^20^ The winning model achieved QWK = 0.884 (95% confidence interval [0.819; 0.949]), and the performance of the second-place finisher was noninferior to that of the winning submission within a margin of 0.02 for ΔQWK. The performances of the other models were inferior to that of the winning submission.^7^ Throughout this paper, models are ordered by decreasing QWK (i.e., “model 1” is the challenge winner).

#### 3.2.2 Binarized classification task

In the binarized classification task of distinguishing between no/mild COVID pneumonia (mRALE ≤ 4, 535 patients) and moderate/severe signs (mRALE > 4, 279 patients), all models performed well in terms of area under the ROC curve (AUC) (Table 2).

**Table 2:** Performance in the task of distinguishing between no/mild and moderate/severe lung involvement in terms of area under the ROC curve (AUC) including the 95% confidence interval (CI), and the percentage of the subgroups experiencing bias in the bias metrics ΔQWK, EOD, and AAOD with respect to the reference groups indicated in Table 1 with an asterisk.

Model	AUC [95% CI]	Percentage of subgroups experiencing bias		
		ΔQWK	EOD	AAOD
1	0.95 [0.931, 0.961]	0% (0/12)	25% (3/12)	25% (3/12)
2	0.95 [0.931, 0.961]	8% (1/12)	0% (0/12)	0% (0/12)
3	0.94 [0.928, 0.960]	8% (1/12)	17% (2/12)	33% (4/12)
4	0.93 [0.914, 0.949]	25% (3/12)	8% (1/12)	17% (2/12)
5	0.93 [0.917, 0.951]	17% (2/12)	25% (3/12)	25% (3/12)
6	0.93 [0.914, 0.948]	25% (3/12)	42% (5/12)	25% (3/12)
7	0.93 [0.910, 0.945]	8% (1/12)	25% (3/12)	33% (4/12)
8	0.92 [0.898, 0.937]	17% (2/12)	25% (3/12)	33% (4/12)
9	0.90 [0.873, 0.917]	17% (2/12)	50% (6/12)	25% (3/12)

### 3.3 Bias Assessment

#### 3.3.1 Estimation problem

**Fig. 2:**
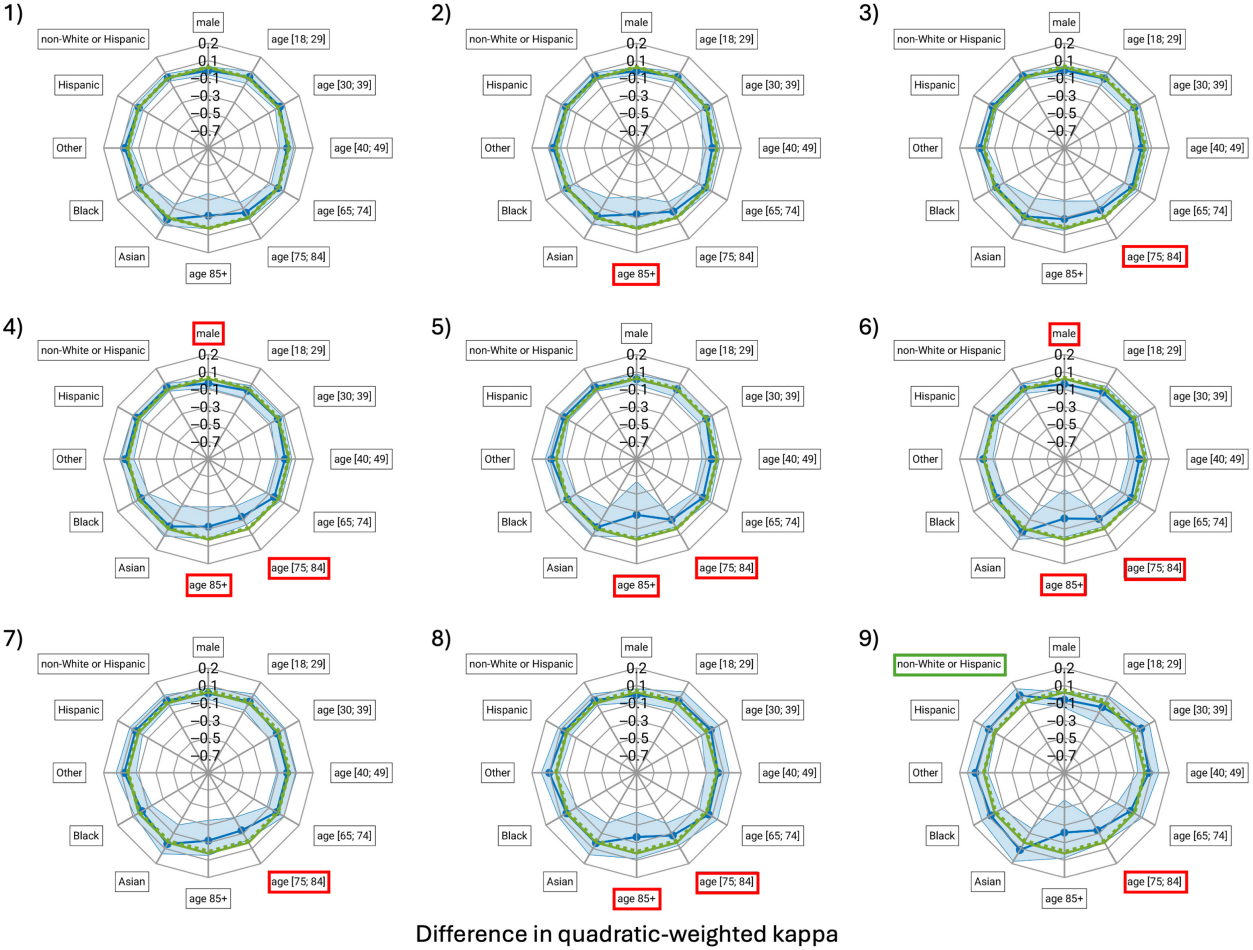
Spider plots of the difference in quadratic-weighted kappa, ΔQWK (blue line with symbols), including 95% confidence interval of this difference (shaded blue region), for the demographic subgroups with respect to the reference groups indicated with an asterisk in Table 1. ΔQWK=0 is indicated for reference (green dashed line). Positive bias is indicated by a green border and negative bias by a red border of the label boxes, respectively. The labels from the top clockwise are: “male,” “age [19; 29],” “age [30; 39],” “age [40, 49],” “age [65; 74],” “age [75; 84],” “age 85+,” “Asian,” “Black,” “Other,” “Hispanic,” and “non-White or Hispanic” (labels slightly abbreviated from those listed in Table 1).

Few differences in QWK among subgroups reached statistical significance, in part due to limited statistical power for the smaller subgroups. Older patients were most likely to receive biased scores from the models, with 8 of 9 models (89%) demonstrating a negative bias for the age groups 75 to 84 and/or 85+ years, indicating worse agreement with the reference mRALE score compared with the reference group of patients aged 50 to 64 years (Fig. 2). The only model for which no bias was identified (within the statistical power of this study) in terms of ΔQWK was the winning submission (Fig. 2). Considering all models, the median number of subgroups experiencing bias in terms of ΔQWK was 2.

#### 3.3.2 Binarized classification problem

The only model for which no bias was identified in terms of both EOD and AAOD was the runner up in the challenge. Of the eight models other than the runner up, most had three subgroups identified as experiencing bias in terms of EOD, with the number of subgroups experiencing bias ranging from 1 (1 model) to 6 (1 model). For these eight models, in terms of AAOD, the median number of subgroups experiencing bias was 3, with numbers ranging from 2 (1 model) to 4 (3 models).

**Fig. 3:**
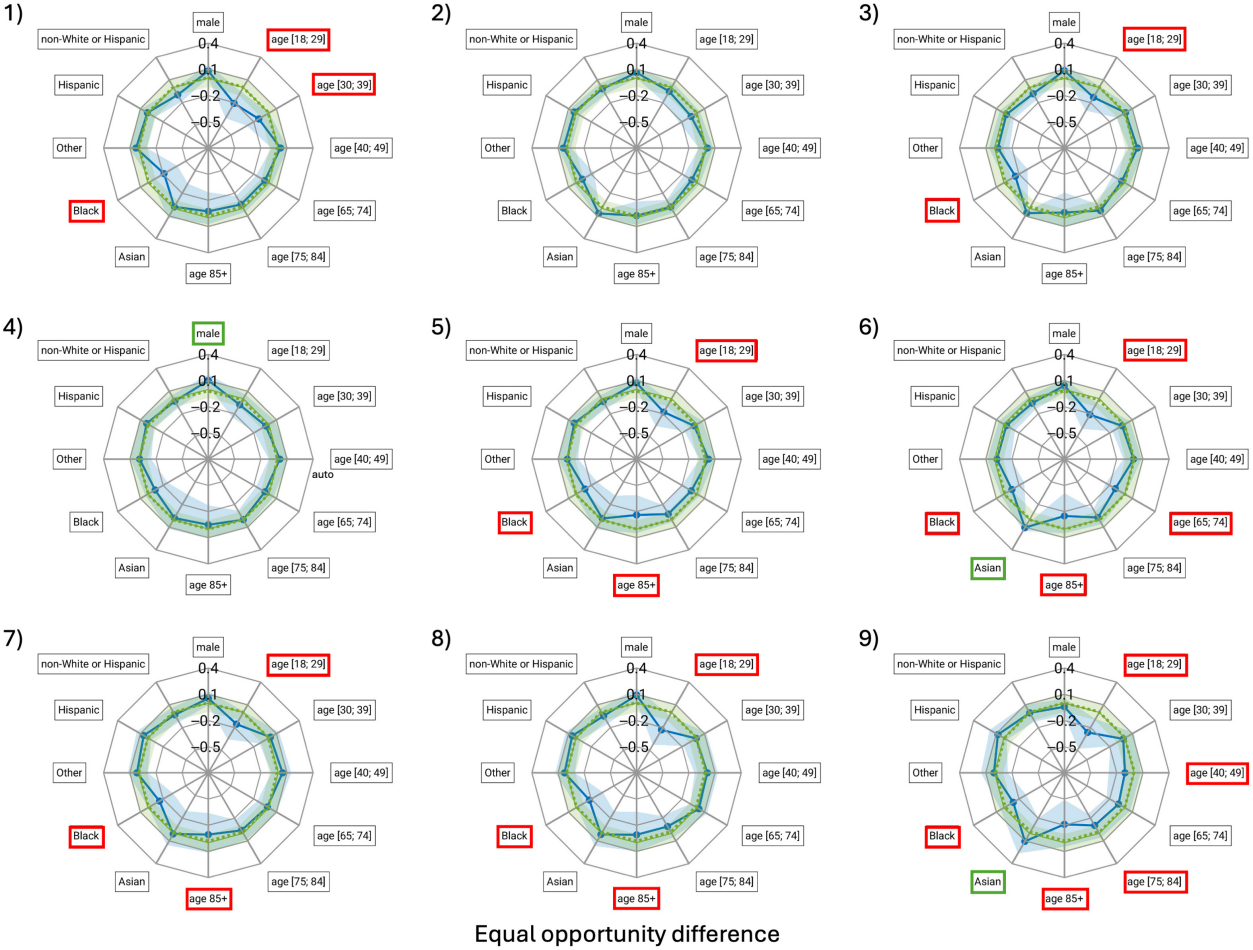
Spider plots of the equal opportunity difference (EOD) including 95% confidence intervals (shaded blue regions) for the demographic subgroups with respect to the reference groups indicated with an asterisk in Table 1. Zero is indicated with a green dashed line, whereas the green shaded regions indicate the acceptable range for the metric, and values outside the range are considered indicative of bias. Positive bias is indicated by a green border and negative bias by a red border of the label boxes, respectively. The labeling of subgroups is the same as for Fig. 2.

The groups most frequently identified as experiencing bias in terms of EOD were the youngest patients (≤29 years, 7 of 9 models), oldest patients (85+ years, 5 of 9 models), and Black/African Americans (7 of 9 models) indicating a bias toward these subgroups to experience lower TPRs (sensitivity) (Fig. 3). The most substantial observed negative bias in terms of EOD was for model 9 with a median of −0.26 (95% confidence interval [−0.48; −0.09]) for patients ≤29 years old. The most substantial positive bias was observed for model 3 with an EOD of 0.11 [0.07; 0.15] for patients of Asian race.

**Fig. 4:**
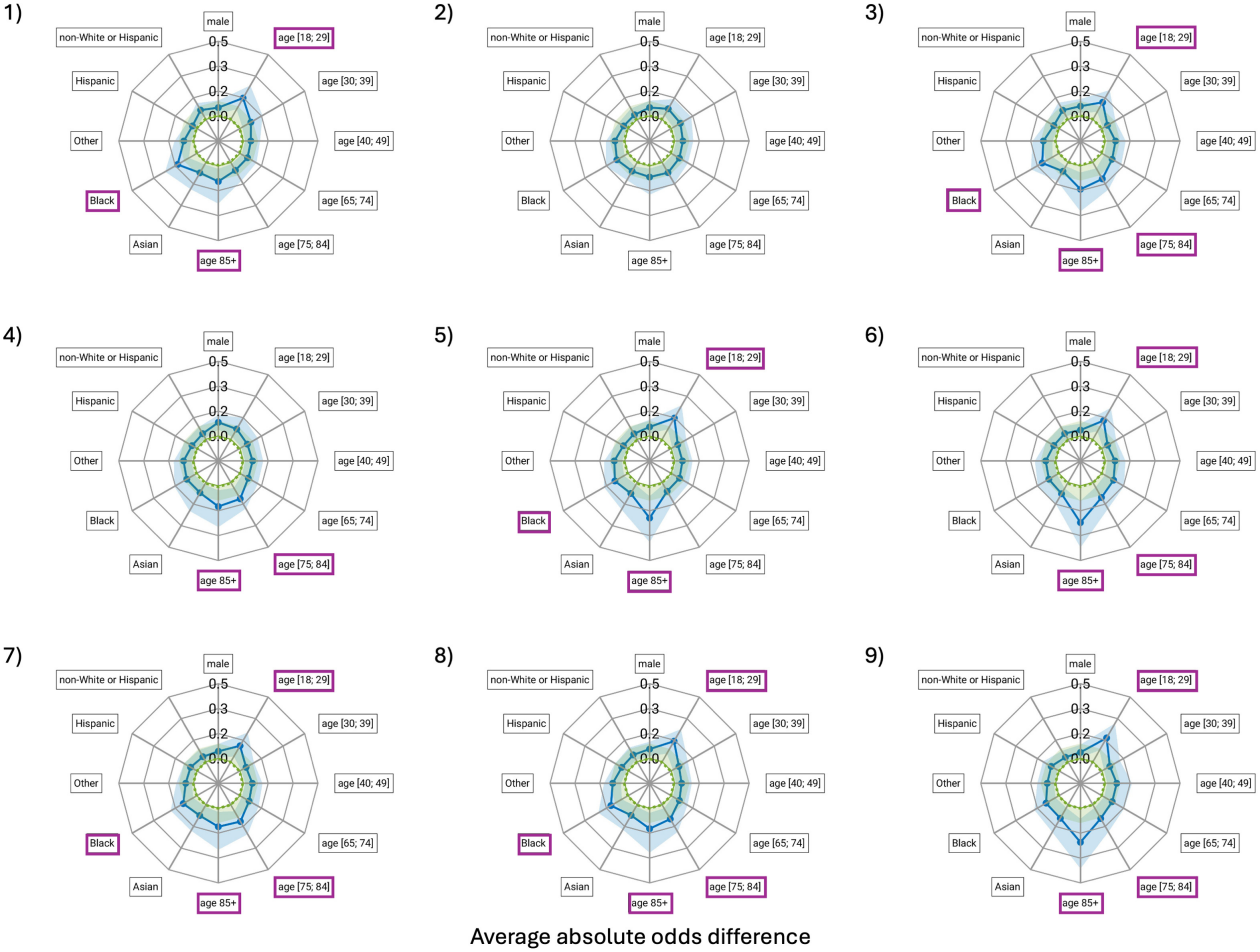
Spider plots of the average absolute odds difference (AAOD) including 95% confidence intervals (shaded blue regions) for the demographic subgroups with respect to the reference groups indicated with an asterisk in Table 1. Zero is indicated with a green dashed line, whereas the green shaded regions indicate the acceptable range for the metric, and values outside the range are considered indicative of bias. As AAOD is based on absolute values, the presence of bias is indicated by a purple border around the label boxes. The labeling of subgroups is the same as for Fig. 2.

The groups most frequently identified as experiencing bias in terms of AAOD were the youngest patients (≤29 years, 7 of 9 models), patients between 75 and 84 years (6 of 9 models), and patients 85+ years (8 of 9 models) (Fig. 4). A higher false-positive rate tended to be the bias for these groups. The largest observed AAOD was for model 6, with a median of 0.26 [0.10; 0.41] for patients ≥85 years old.

## 4 Discussion and Conclusion

The MIDRC mRALE Mastermind Challenge demonstrated that AI/ML models can achieve high performance in the automated assessment of COVID-19 severity using portable CXRs, with most models exhibiting good agreement with the reference standard. The range of QWK values, from 0.74 to 0.88, underscores the overall accuracy of the models in predicting mRALE scores. However, this study also highlighted that high performance does not necessarily equate to fairness across demographic subgroups. The observed biases in some models suggest that even well-performing models can inadvertently disadvantage certain groups, underscoring the need for rigorous bias assessment alongside performance evaluation.

All participants used the MIDRC-provided annotated training set as part of their model development and training. Participants employed a range of training strategies, with several using additional datasets or transfer learning from pre-trained models, such as those from the RSNA Pneumonia Detection Challenge^21^ (model 1), CheXpert^22^ (model 4), TorchXrayVision^23^ (model 6), and ImageNet-22K^24^ (model 8). Interestingly, biases among models varied, even when they were trained, in part, on the same data provided by MIDRC and evaluated with the same reference standard.

In the study presented here, only performance on the test set was considered. Although participants did not have access to either the validation or the test sets, they were allowed multiple submissions during the validation phase to fine-tune their models. For that reason, performance on the test set is a more reliable indicator of model performance, generalization, and bias. The main limitations of this study include the modest size of the test set (814 patients) and the imbalance with respect to disease severity in the training, validation, and test sets. Although this reflects the realities of clinical practice during the pandemic, it complicates model development and evaluation. The cohort comprising the validation and test sets for the challenge was sampled from not yet publicly available cases ingested into MIDRC and was selected to approximately match the CDC demographic distributions for COVID-positive cases at the time. Moreover, the cohort was then stratified into a validation and test set matched for demographics, disease severity, and the presence of medical devices such as EKG leads or breathing tubes, and triply annotated. The provided training set, on the other hand, contained all portable CXR exams available at the time in the MIDRC open data commons, matching the inclusion criteria^7^ (1198 patients, 2072 imaging studies). All exams were singly annotated, and all annotations (mRALE scores) were made available. As such, the training dataset differed somewhat from the validation and test sets in both demographic distribution and disease severity. The training set included patients with slightly more severe COVID-19, with only 20% of cases scoring an mRALE of 0 (no apparent disease), compared with 26% in the test set, and 45% receiving mRALE scores of 4 or less (no/mild disease), compared with 66% in the test set. In addition, the training cohort had proportionally fewer younger patients (∼8% < 30 years old) and fewer Black or African Americans (at 8% versus 17% in the validation and test sets).

Another limitation was that although 95% confidence intervals were provided (Figs. 3 and 4), only the median value of the EOD and AAOD metrics was used to label subgroups as experiencing bias. If a more stringent criterion was applied that the entire 95% confidence lie within the “allowed” range ([−0.1; 0.1] and [0; 0.1] for EOD and AAOD, respectively), generally bias could only be excluded with respect to ethnicity and intersectional race/ethnicity.

This study also underscores the challenge of evaluating bias with limited statistical power in smaller subgroups. For example, the smaller sample of patients aged ≥85 years (N=33) may not have been as representative as the larger samples for other subgroups. This highlights the need for larger and more diverse datasets to enable more robust bias evaluations. The metrics used in this study—QWK differences, EOD, and AAOD—each offer insights into different aspects of model performance, but they also have limitations. For example, EOD focuses on TPRs but does not account for false positives, which are a key concern in many medical applications. AAOD, which incorporates both TPR and FPRs, provides a more balanced view of fairness but can be harder to interpret. In addition, although these metrics are designed to detect disparities, they may be influenced by factors such as disease prevalence within subgroups. For instance, EOD is theoretically independent of disease prevalence, but in practice, if one subgroup has a significantly higher prevalence of the disease, the model may struggle to maintain the same TPR across groups, leading to apparent bias.

In practice, there is often an implicit assumption of a Pareto inequality^25^—that improvements in fairness or equity for some subgroups may require trade-offs in predictive performance, or vice versa. By systematically benchmarking submissions from the mRALE Mastermind Challenge, our study examined these potential trade-offs in the top-performing model from each team participating in the test phase. The winning submission maintained high accuracy, and we did not find statistically significant differences in subgroup agreement with the reference standard, although statistically significant differences were observed for some groups in terms of equal opportunity difference and average absolute odds difference. Conversely, for the runner-up, no statistically significant differences were detected according to these two fairness metrics for the binarized prediction task, but a statistically significant difference was observed for a single subgroup in agreement with the reference standard. These findings suggest that it may be possible to approach Pareto-efficient solutions—where both accuracy and fairness are achieved simultaneously—in real-world AI applications, providing insight for future model development and evaluation standards. One should note, however, that although a biased model with high overall performance may raise concerns about fairness, it could still be a suitable choice in scenarios where its limitations are well understood, and its use can be targeted appropriately. For instance, if a model exhibits strong predictive accuracy for specific subgroups but underperforms for others, it might still provide value when deployed exclusively for the subgroups where it excels, provided that these biases are transparently documented and accounted for in clinical decision-making.

Overall, the MIDRC mRALE Mastermind Challenge demonstrated encouraging performance in the automated assessment of COVID-19 severity. Variations in bias highlight the importance of ongoing bias evaluation in AI-driven healthcare. This finding underscores the potential for developing models that are both accurate and equitable, enabling AI solutions that promote fairer healthcare without sacrificing performance. Moving forward, the inclusion of more comprehensive bias metrics, as well as a focus on intersectionality (i.e., how different demographic factors such as age, race, and ethnicity interact), will be essential for developing AI/ML models that are not only accurate but also equitable. In addition, integrating strategies for bias mitigation during the model training phase, rather than assessing bias post-hoc, could further enhance the fairness of AI models in medical imaging.

## Data Availability

Training data: data.midrc.org. Instructions for downloading the training data and mRALE annotations: https://github.com/MIDRC/MIDRC_Grand_Challenges/tree/main/Challenge_2023_mRALE%20Mastermind Challenge submissions’ model code, weights, and descriptions: https://github.com/MIDRC/MIDRC_Grand_Challenges/tree/main/Challenge_2023_mRALE%20Mastermind Generalized version of the code for identifying subgroup performance discrepancies and generating spider plots: https://github.com/MIDRC/MIDRC_MELODY
